# Unique Case of Gastroenteritis Presenting as Pneumatosis Intestinalis With Emphysematous Gastritis and Portal Vein Gas: Do Not Take Gastroenteritis Lightly

**DOI:** 10.7759/cureus.8765

**Published:** 2020-06-22

**Authors:** Asif Mehmood, Rajesh Essrani, Swetha Parvataneni, Umair Iqbal

**Affiliations:** 1 Internal Medicine, Geisinger Medical Center, Danville, USA; 2 Internal Medicine, Abington Hospital - Jefferson Health, Abington, USA; 3 Internal Medicine, Lehigh Valley Health Network, Allentown, USA; 4 Internal Medicine, Geisinger Health System, Lewistown, USA; 5 Internal Medicine, Geisinger Commonwealth School of Medicine, Danville, USA

**Keywords:** gastroenteritis, pneumatosis intestinalis, emphysematous gastritis

## Abstract

Pneumatosis intestinalis (PI) consists of multiple, thin, gas-filled cysts in the wall of the gastrointestinal (GI) tract. It is an uncommon entity that can involve any gastrointestinal site from the stomach to the rectum. Isolated stomach involvement is rare. PI can represent a broad spectrum of diseases with variable prognoses. We present the case of a patient who was admitted with gastroenteritis-like symptoms. He remained hemodynamically stable, and on further imaging with contrast-enhanced computed tomography of the abdomen and pelvis, air was found in the portal vein and gastric wall, with minimal thickening of the proximal small bowel concerning for emphysematous gastroenteritis. Further workup results were negative, including blood cultures, stool studies, Clostridium difficile toxins, and lactic acid levels. The patient was managed nonoperatively and recovered without serious complications. Our case is unique in terms of the presence of air in the portal vein, which would otherwise suggest the possible spread of infection across the bowel wall.

## Introduction

Pneumatosis intestinalis (PI) refers to the presence of multiple, thin, gas-filled cysts in the wall of the gastrointestinal (GI) tract. It is a rare condition made evident by the increased use of radiographic imaging and can involve any site along the gastrointestinal tract. The occurrence of pneumatosis is distributed as follows: 46% in the colon, 27% in the small bowl, 7% in both the small and large intestine, and 5% in the stomach [1]. Primary PI is idiopathic and rare. Secondary PI is associated with numerous gastrointestinal conditions like intestinal obstruction, ischemic bowel disease, and necrotizing enterocolitis in premature infants. It can also occur with other conditions like chronic obstructive pulmonary disease, acquired immunodeficiency syndrome, bacterial or viral infection, or adverse events related to chemotherapy or other drugs [2-9]. Radiologically, PI can take two forms: bubble-like and continuous [10-11].

## Case presentation

**Figure 1:**
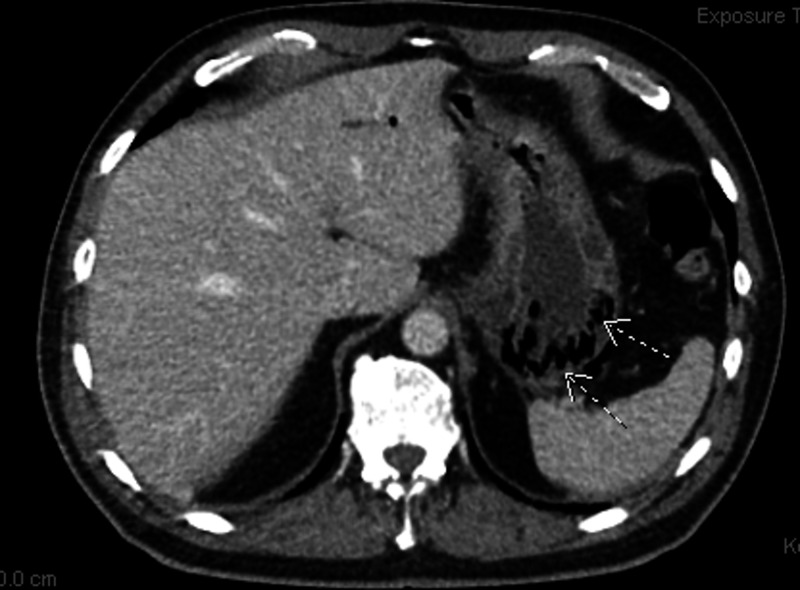
CT scan axial view showing gas in the stomach walls CT: computed tomography

An 87-year-old man presented with multiple episodes of non-bloody, watery diarrhea associated with nausea and epigastric pain for two days. He had a past medical history of hypertension, hyperlipidemia, and coronary artery disease with bypass surgery. He denied any fever, vomiting, sick contact, recent travel, or recent antibiotic use. On physical exam, his blood pressure was 146/90 mmHg, pulse rate was 90 beats per minute, respiratory rate was 20 breaths per minute, and temperature was 100.8°F. He was non-toxic-appearing without acute distress, mild epigastric tenderness with hypoactive bowel sounds was noted, without rebound tenderness or guarding. Normal vesicular breathing without any crackles or wheezing on chest exam. Laboratory findings showed normal blood counts except leukocytosis of 14,000/ml, elevated urea blood levels of 30 mmol/L, and creatinine level of 1.3 mg/dl. A contrast-enhanced computed tomography (CECT) scan of the abdomen and pelvis showed air in the portal vein and gastric wall, with minimal thickening of the proximal small bowel, which was concerning for emphysematous gastroenteritis (Figure 1).

Further workup results were negative, including blood cultures, stool studies, Clostridium difficile toxins, and lactic acid levels. He was admitted to the intensive care unit (ICU) for closer monitoring and evaluated by surgery, gastroenterology, and infectious disease specialists. His care team decided to treat the patient nonoperatively based on his hemodynamic stability and a benign abdominal exam. The patient was placed on a “nil per os” (i.e., nothing by mouth) order and received intravenous (IV) fluids and IV antibiotics, including ciprofloxacin and metronidazole. With no other identifiable cause of PI, the patient’s presentation was ultimately thought to represent a mild bacterial infection yielding minimal clues to the definitive diagnosis aside from radiographic data. The patient recovered well with nonoperative management and was discharged on oral antibiotics after a few days, with plans to follow up with outpatient CT scan imaging.

## Discussion

The significance of PI depends on the severity and nature of the underlying condition [12]. Therefore, it can present a wide spectrum of conditions and outcomes, which can range from very benign diseases to abdominal sepsis, bowel infarction, and death. Many factors are involved in the pathogenesis of PI. Its mechanical component is important: gas traverses the mural portion of the bowel; this can be triggered by minor damage to the mucosa caused by inflammation or necrosis and can also occur in the setting of increased intraabdominal pressure, by direct gas diffusion through the mucosa [13-14]. Another important aspect is the origin of the gas; there is always some amount of intramural gas present in the human bowel, but bacterial overgrowth and invasion of the bowel wall can result in excess gas production favoring the formation of PI [15]. A systemic analysis of even benign presentation can be very helpful to avoid complications. From a clinical point of view, an urgent distinction between benign and life-threatening causes is needed.

In our case, based on radiographic findings of air within the bowel on CT scan, the patient was diagnosed with emphysematous gastroenteritis. With no other exact cause of PI, with further workup being negative. including culture data, the patient’s presentation was ultimately thought to represent a mild bacterial infection. The patient was treated conservatively and his gastrointestinal symptoms resolved without sequelae, further reinforcing the diagnosis. Our case is very unique in terms of the presence of air in the portal vein, which kind of points towards the spread of infection across the bowel wall but without an expected grave outcome [16].

## Conclusions

Emphysematous gastritis with air in the portal vein is rare in the setting of underlying gastroenteritis, a common disease worldwide. The diagnosis can mimic viral gastroenteritis and can be easy to miss, especially if the initial presentation is benign. A high index of suspicion with a low threshold for scanning can be lifesaving.
